# A Novel Model of IgE-Mediated Passive Pulmonary Anaphylaxis in Rats

**DOI:** 10.1371/journal.pone.0116166

**Published:** 2014-12-26

**Authors:** Eva Wex, Eva Thaler, Sylvia Blum, David Lamb

**Affiliations:** Respiratory Diseases Research, Boehringer Ingelheim Pharma GmbH & Co. KG, Biberach, Germany; French National Centre for Scientific Research, France

## Abstract

Mast cells are central effector cells in allergic asthma and are augmented in the airways of asthma patients. Attenuating mast cell degranulation and with it the early asthmatic response is an important intervention point to inhibit bronchoconstriction, plasma exudation and tissue oedema formation. To validate the efficacy of novel pharmacological interventions, appropriate and practicable *in vivo* models reflecting mast cell-dependent mechanisms in the lung, are missing. Thus, we developed a novel model of passive pulmonary anaphylaxis in rats. Rats were passively sensitized by concurrent intratracheal and intradermal (ear) application of an anti-DNP IgE antibody. Intravenous application of the antigen, DNP-BSA in combination with Evans blue dye, led to mast cell degranulation in both tissues. Quantification of mast cell degranulation in the lung was determined by (1) mediator release into bronchoalveolar lavage, (2) extravasation of Evans blue dye into tracheal and bronchial lung tissue and (3) invasive measurement of antigen-induced bronchoconstriction. Quantification of mast cell degranulation in the ear was determined by extravasation of Evans blue dye into ear tissue. We pharmacologically validated our model using the SYK inhibitor Fostamatinib, the H_1_-receptor antagonist Desloratadine, the mast cell stabilizer disodium cromoglycate (DSCG) and the β_2_-adrenergic receptor agonist Formoterol. Fostamatinib was equally efficacious in lung and ear. Desloratadine effectively inhibited bronchoconstriction and ear vascular leakage, but was less effective against pulmonary vascular leakage, perhaps reflecting the differing roles for histamine receptor sub-types. DSCG attenuated both vascular leakage in the lung and bronchoconstriction, but with a very short duration of action. As an inhaled approach, Formoterol was more effective in the lung than in the ear. This model of passive pulmonary anaphylaxis provides a tissue relevant readout of early mast cell activity and pharmacological benchmarking broadly reflects responses observed in patients with asthma.

## Introduction

Asthma is an inflammatory airway disorder with increasing prevalence currently affecting 235 million people worldwide [1]. The characteristic chronic inflammation of the airways seen in asthma significantly contributes to many features of the disease, including variable airflow obstruction and bronchial hyperresponsiveness which cause recurrent episodes of wheezing, breathlessness, chest tightness, and coughing [2]. According to the Global Initiative for Asthma (GINA) clinical manifestations of asthma can be controlled with appropriate treatment, but in spite of the considerable progress that has been made in the past decade there are still many patients who have not benefited from advances in asthma treatment [3]. Thus, novel and innovative approaches are urgently needed to further improve asthma control.

Many inflammatory cells contribute to the chronic inflammation seen in asthma including infiltrating eosinophils and T-helper 2 (Th2) lymphocytes, as well as resident mast cells and dendritic cells [4]. Mast cells are the central effector cells in allergic asthma and are augmented in the airways of asthma patients [5]. The inhalation of allergens by patients with allergic asthma leads to the cross-linking of FcRI-bound allergen-specific IgE, thereby inducing mast cell degranulation and with it the immediate release of a variety of preformed mediators, including histamine, tryptase, TNFα and IL-4. Furthermore, new synthesis of arachidonic acid metabolites like, cysteinyl leukotrienes (LTC_4_, LTD_4_, and LTE_4_), and prostaglandin D_2_ is stimulated [6]. The antigen-induced release of these mediators results in the early asthmatic response (EAR) which is characterized by the contraction of airway smooth muscle cells and an increase in vascular permeability [7]. Thus, attenuating mast cell degranulation and with it the EAR is an important intervention point to inhibit bronchoconstriction and plasma exudation from the bronchial microvasculature and the formation of tissue oedema.

Currently available asthma medications include short-acting and long-acting inhaled β_2_-agonists, inhaled corticosteroids, cromones, methylxanthines, leukotriene inhibitors, and an anti-IgE monoclonal antibody, all modifying aspects of allergen-induced responses [8]. Asthma research is striving to develop new and effective treatment options to further improve symptom control as the most important treatment outcome. To validate pharmacological interventions pre-clinical *in vivo* models are required which on the one hand closely reflect aspects of the pathophysiology found in asthma and on the other hand are efficient, easy to reproduce, and reliable. Available preclinical *in vivo* models involve models of local or systemic active or passive anaphylaxis. Active anaphylaxis is induced by the administration of antigens to animals that have been sensitized with the same agent before. In contrast, passive anaphylaxis is most often stimulated by antigen challenge in animals that have received injections of antigen-specific IgE antibodies [9]. However, most available models suffer from shortcomings including the fact that they are either not performed in the target organ (e.g. the lung), are time-consuming or show a high variability, which make them inappropriate to screen for new therapeutic treatment options.

Thus, our aim was to develop a novel passive pulmonary anaphylaxis model in the rat which reflects aspects of the EAR seen in asthmatic patients and which is timesaving, technically easily manageable, and shows high reproducibility. For this purpose we instilled rats with a single intratracheal dose of an anti-DNP IgE antibody and one day later pulmonary anaphylaxis was induced by the intravenous application of the antigen, DNP-BSA, in combination with Evans blue. Our model reflects pathophysiological features of the EAR including IgE-induced mast cell degranulation and mediator release, the formation of tissue oedema and bronchoconstriction as determined by the measurement of mast cell mediators in the bronchoalveolar lavage fluid (BALF), quantification of Evans blue leakage into tracheal and bronchial tissue and invasive lung function measurement. Furthermore, the well-established method of passive cutaneous anaphylaxis in ear tissue can be performed simultaneously in the same animals further reducing animal numbers and saving time [10]. To validate the model we tested different emerging or clinically relevant asthma medications including the SYK inhibitor Fostamatinib, the H_1_-receptor antagonist Desloratadine, the mast cell stabilizer disodium cromoglycate (DSCG) and the long-acting β_2_-adrenergic receptor agonist Formoterol.

## Materials and Methods

### 1.1. Reagents and test compounds

Anti-DNP IgE antibody (monoclonal anti-dinitrophenyl antibody produced in mouse, IgE isotype, clone SPE-7), Evans blue, disodium cromoglycate, Desloratadine and formamide were purchased from Sigma Aldrich (Sigma-Aldrich, Co., St. Louis, MO). DNP-BSA (DNP-Albumin Conjugate, Bovine) was from Calbiochem (EMD Chemicals, Inc., San Diego, CA). Formoterol fumarate and Fostamatinib were synthesized at the Department of Chemical Research, Boehringer Ingelheim Pharma GmbH and Co. KG (Biberach, Germany). Hydroxyethylcellulose was from Merck (Merck Schuchardt OHG, Hohenbrunn, Germany). Tween20 was purchased from Acros Organics (New Jersey, USA). Narcoren was from Merial GmbH (Halbergmoos, Germany). Isoflurane (Forene) was from Abbott (Wiesbaden, Germany). Ketamine hydrochloride (Ketavet) was purchased from Pfizer Pharmacia GmbH (Berlin, Germany). Pancuronium bromide (Pancuronium Inresa) was from Inresa Arzneimittel GmbH (Freiburg, Germany).

### 1.2. Animals

Adult, test-naïve, female albino Wistar Han IGS rats (Crl:WI(Han); 11–15 weeks; approx. 230 g) were purchased from Charles River (Charles River Laboratories International Inc., Sulzfeld, Germany). Animals were kept in rooms maintained at constant temperature (22°C±2°C) and humidity (60%±15%) under a 12 h light-dark cycle. The animals were housed in groups of two in isolated ventilates cages and allowed free access to water and standard food. All animal experimentation was conducted in accordance with German national guidelines and legal regulations and approved by the ethical committee Regierungspräsidium Tübingen (Germany) (Permit Number: 10-008). All surgery was performed under sodium pentobarbital and ketamine hydrochloride anaesthesia, and all efforts were made to minimize suffering. Animals were constantly monitored during induction and maintenance of anaesthesia, and the level of narcotic adjusted to maintain optimal depth of anaesthesia. Humane endpoints were used throughout the study and no animals died without humane intervention. Three animals died under anaesthesia.

### 1.3. Compound application

Fostamatinib was dissolved in 0.5% hydroxyethylcellulose containing 0.01% Tween20 and applied by oral gavage one hour prior to the antigen. Desloratadine was dissolved in 0.5% hydroxyethylcellulose and applied by oral gavage one hour prior to the antigen. Disodium cromoglycate was dissolved in 0.9% saline and administered intravenously via the tail vein together with the antigen. Formoterol fumarate was dissolved in distilled water and aerosolized with a jet nebulizer (Parimaster compressor with Pari LL nebulizer). Whole body exposure of rats to Formoterol was conducted for 5 min in a custom made cylindrical 32 L Perspex box 30 min before administration of the antigen.

### 1.4. Passive pulmonary and cutaneous anaphylaxis

For administration of the monoclonal anti-DNP IgE antibody rats were short-time anesthetized with Isoflurane (3–4% v/v). For the intratracheal administration of the antibody the animals were fixed in a supine position and 200 µl of antibody (50 µg/ml in PBS) was administered into the lung of each animal. Furthermore, each animal received 10 µl of antibody (1 µg/ml in PBS) intradermally into the right ear. To determine the negative signal for the passive pulmonary anaphylaxis separate animals received 200 µl PBS into the lung. To determine the negative signal for the ear passive cutaneous anaphylaxis each animal served as its own negative control and received 10 µl PBS into the left ear. 24 h later animals were short-time anesthetized with Isoflurane (3–4% v/v). 1 ml of 1 mg DNP-BSA diluted in 0.9% saline with 1% Evans blue was injected into the tail vein. Thirty minutes after the DNP-BSA challenge rats were euthanized by intraperitoneal pentobarbital injection (400 mg/kg Narcoren). The respiratory tract, including the larynx, trachea, and lung was removed. The thyroid gland was removed and the lung was dissected free of the parenchyma. Ear tissue biopsies were taken from both ears using a tissue punch (8 mm diameter). The Evans blue was extracted by incubating the trachea together with the bronchial tissue in 2.5 ml and the ear tissue in 0.5 ml of formamide at 65°C overnight with gentle agitation (450 rpm). The next day the absorbance in the supernatant was measured at 620 nm using a spectrophotometer.

### 1.5. Measurement of antigen-induced bronchoconstriction

Rats were anaesthetized by intraperitoneal injection of Narcoren (60 mg/kg at a volume of 1 ml/kg) and subsequent intramuscular injection of ketamine hydrochloride (100 mg/kg at a volume of 1 ml/kg). An intravenous cannula was inserted in the tail vein and fixed. After tracheostomy a tracheal cannula was inserted and fixed by a ligature. The tracheal cannula was connected to an integrated ventilation and lung function measurement device (Scireq flexiVent, EMKA Technologies, Paris, France) with the software version 7.2. The spontaneous respiration was suppressed by intravenous administration of pancuronium bromide (0.8 mg/kg at a volume of 1 ml/kg). Ventilation was conducted with a tidal volume of 10 ml/kg, a breathing frequency of 90 breaths/minute and a positive end expiratory pressure of 3 cmH_2_O using the template “FlexiVent FC-Rat Default-rel B”. After recruiting total lung capacity (30 cmH_2_O) baseline airway resistance, elastance and compliance were determined. To induce bronchoconstriction a bolus of 1 mg DNP-BSA diluted in 0.9% saline with 1% Evans blue in a total volume of 1 ml was injected into the tail vein within 15 sec. Maximal bronchoconstriction within 5 min after DNP-BSA application was determined. After lung function measurement animals were euthanized by intravenous pentobarbital injection (200 mg/kg Narcoren). For data analysis the difference between the basis value and the maximal value within 5 min after antigen administration was calculated.

### 1.6. Bronchoalveolar lavage and determination of mediators in BALF

After intravenous DNP-BSA application rats were euthanized by intraperitoneal pentobarbital injection (400 mg/kg Narcoren). Trachea was exposed and a tracheal tube was fixed with a ligature just beneath the larynx. Bronchoalveolar lavage was performed with 4 ml PBS. Lavage was centrifuged for 5 min at 1,500 rpm and supernatant was stored at −80°C for assessment of mediators. Histamine content was measured using an ELISA kit from Biotrend Chemikalien GmbH (Köln, Germany). Tryptase was determined using an ELISA kit from USCN Life Science Inc. (Wuhan, Hubei, PCR). The cysteinyl leukotrienes were determined using the Cysteinyl Leokotriene Express EIA Kit from Cayman Chemical (Ann Harbor, MI).

### 1.7. Statistical analysis

**Figure 1:**
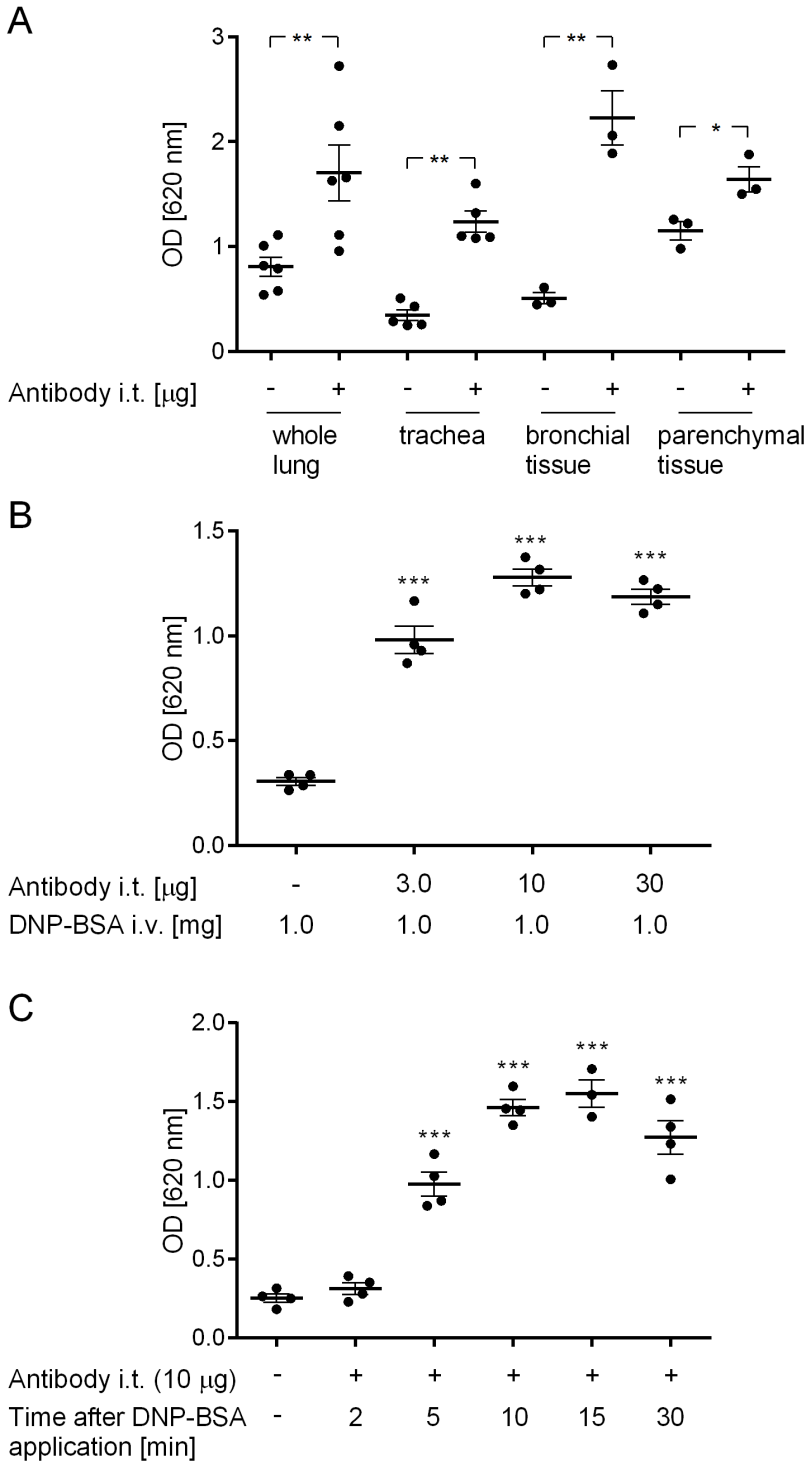
Optimization of experimental conditions to induce lung oedema formation. (A) Evans blue dye recovery from whole lung, the trachea, the bronchial and parenchymal tissue was compared. (B) The anti-DNP IgE antibody dose was titrated. (C) Evans blue dye recovery at different times after DNP-BSA application was tested. Statistical significance was calculated by t-test in (A) and by 1-way ANOVA with subsequent Dunnett’s multiple comparisons test in (B) and (C). *p <0.05, **p <0.01 and ***p <0.001 represent significant differences compared with rats that did not receive the anti-DNP IgE antibody. Data of individual animals ± SEM are shown (n = 3–6 animals per group).

Data were presented as either individual values or as mean ± S.E.M. Statistical differences between two groups in Fig. 1A were compared using a parametric two-tailed t-test. More than two groups were compared with the one-way variance analysis (ANOVA) followed by the Dunnett's test to correct for multiple comparisons. The limit of the significance was taken as *p* values less than 0.05 (*p* <0.05). The half-maximal effective dose (ED_50_) values of the compounds were calculated with 95% confidence intervals by a nonlinear regression analysis. The percentage inhibition was calculated from the mean value in the positive group according to: % inhibition = 100-(Y/K1)*100. With K1 being the mean value of the vehicle-treated negative control group subtracted from the mean value of the vehicle-treated positive control group and Y being the mean value of the vehicle-treated negative control group subtracted from the mean value of the respective compound-treated group. These tests were performed using GraphPad Prism version 6.01 for Windows, GraphPad Software, La Jolla, California, USA, “www.graphpad.com”.

## Results

### 2.1. Optimization of experimental conditions to induce lung oedema formation

Initial experiments were performed in order to establish the ideal Evans blue dye recovery protocol and the optimal anti-DNP IgE antibody dose which provided the largest assay window. In a first step Evans blue dye recovery was compared with signal in the whole lung, the trachea, the bronchial and parenchymal tissue. The trachea and the bronchial tissue showed the most preferable signal-to-noise ratio and variability (p <0.01; Fig. 1A). In an attempt to reduce the signal-to-noise ratio, we perfused the pulmonary vasculature with saline prior to dye extraction, however, this had no impact on the background signal (data not shown). Intratracheally applied anti-DNP IgE antibody prior to allergen challenge resulted in a dose-dependent increase in the amount of dye extracted from the trachea and the bronchial tissue above the negative control value, which plateaued at 10 µg (p <0.001; Fig. 1B), which therefore was chosen as the standard dose for further experiments. In a separate experiment, a bolus instillation of IgE into the trachea was compared with a microspray delivery, however, no difference in either the signal nor distribution of extravasated dye was observed (data not shown). Evans blue dye leakage into the lung tissue was found to occur in a time-dependent manner after i.v. administration of the antigen, which was significantly increased above the background signal 5 minutes after intravenous application of the antigen (p <0.001; Fig. 1C). The signal reached a plateau 10 minutes after application of the antigen and remained constant thereafter. The 30 minute time point was adopted for future experiments.

### 2.2. Determination of mast cell mediators in the bronchoalveolar lavage fluid

**Figure 2:**
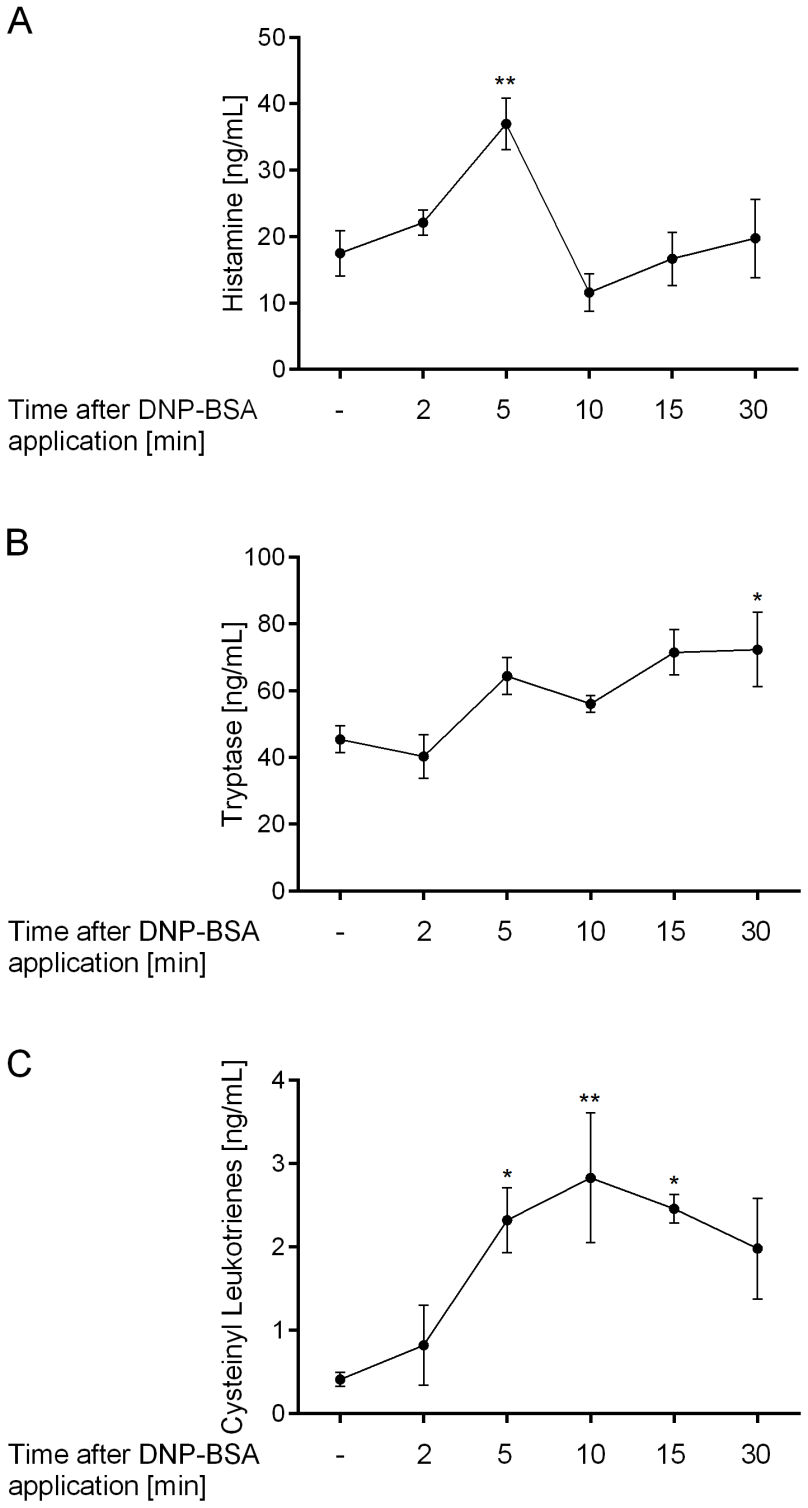
Measurement of antigen-induced mast cell mediator release into bronchoalveolar lavage fluid of passively sensitized rats. Rats were passively sensitized by intratracheal application with 10 µg of anti-DNP IgE antibody. The next day mast cell degranulation was induced by intravenous DNP-BSA application. Rats were lavaged once with 4 ml PBS at 0, 2, 5, 10, 15 and 30 minutes after DNP-BSA application. (A) Histamine, (B) tryptase and (C) cysteinyl leukotrienes were measured in the bronchoalveolar lavage fluid. Statistical significance was calculated by 1-way ANOVA with subsequent Dunnett’s multiple comparisons test compared to rats that only received DNP-BSA but no anti-DNP IgE antibody with *p<0.05 and **p<0.01. Data are given as mean ± SEM of (n = 3–5 animals per group).

To determine the antigen-induced release of mast cell mediators the lungs of the rats were lavaged at different times after application of DNP-BSA and a number of mediators were measured. Histamine levels were significantly increased at 5 min after antigen application and were back to background levels already 10 minutes after antigen application (p <0.01; Fig. 2A). Tryptase levels in the BALF continuously increased over time and were significantly increased only 30 minutes after DNP-BSA administration (p <0.05; Fig. 2B). Cysteinyl leukotriene levels were significantly increased above the background levels at 5 minutes after antigen administration and stayed elevated at 10 and 15 minutes (p <0.05 at 5 and 15 minutes and p <0.01 at 10 minutes; Fig. 2C).

### 2.3. Determination of antigen-induced changes in lung function parameters

**Figure 3:**
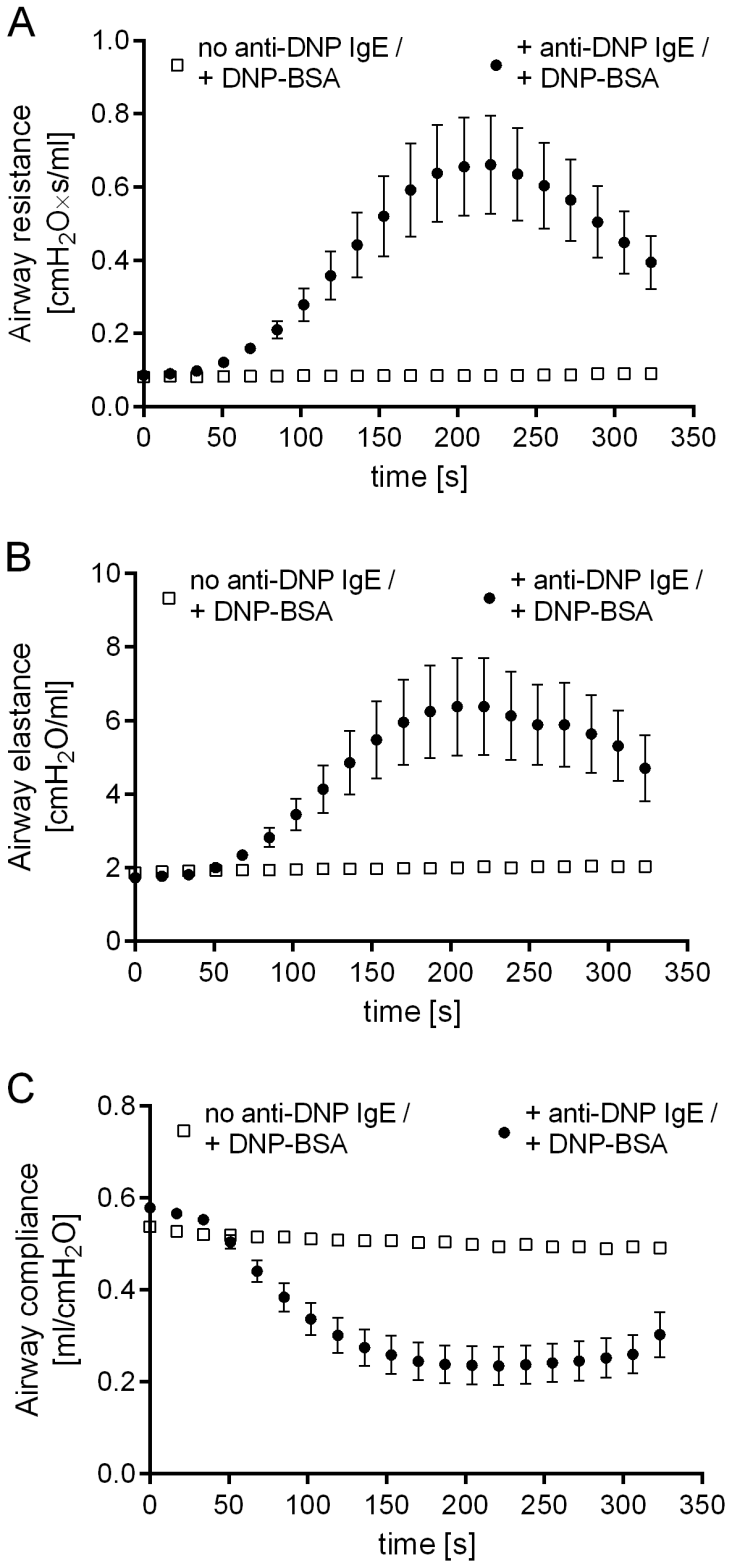
Invasive measurement of antigen-induced bronchoconstriction in anaesthetised rats. Rats were passively sensitized by intratracheal application with 10 µg of anti-DNP IgE antibody. The next day rats were anaesthetised and changes in airway (A) resistance, (B) elastance and (C) compliance within 5 minutes after intravenous DNP-BSA application were recorded. Data are given as mean ± SEM of (n = 4 animals in the negative group and n  = 12 animals in the anti-DNP IgE antibody-treated group).

Using the same parameters as determined in the lung oedema experiments described above, the antigen-induced bronchoconstriction was monitored in a further set of experiments. Changes in airway resistance, elastance and compliance within 5 minutes after DNP-BSA application were recorded. Airway resistance and elastance gradually increased after intravenous DNP-BSA application reaching a maximum approximately 200 seconds after DNP-BSA application (Fig. 3A and 3B). Airway compliance gradually decreased after DNP-BSA application reaching a plateau approximately 170 seconds after application of the antigen (Fig. 3C). No change in any lung function parameter was observed in the control animals that received the DNP-BSA, but not the anti-DNP IgE.

### 2.4. Pharmacological inhibition of lung oedema formation

**Figure 4:**
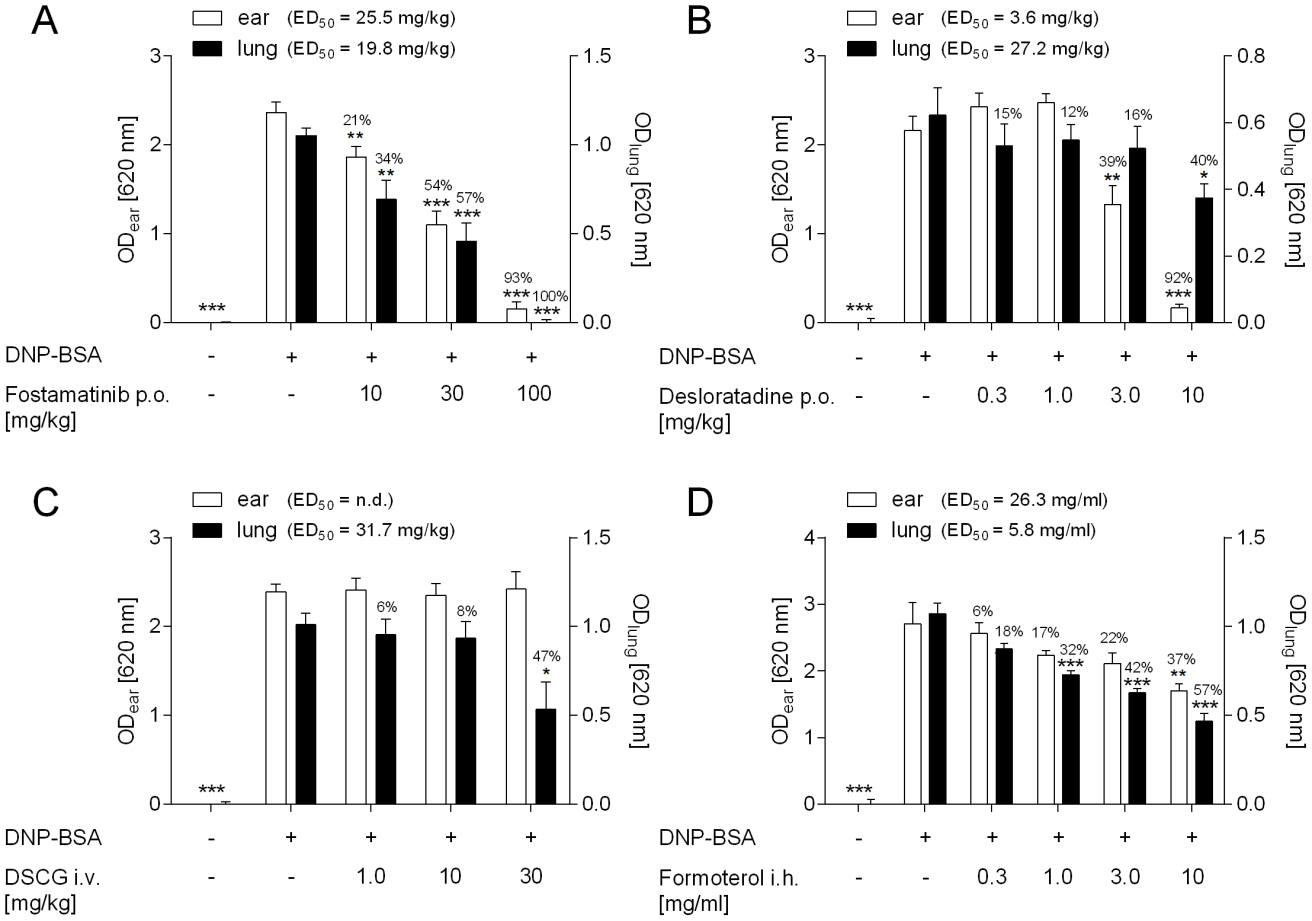
Pharmacological inhibition of antigen-induced lung and ear oedema formation. Rats were passively sensitized by intratracheal and intradermal application of the anti-DNP IgE antibody into the lung and ear. The next day rats were treated with either (A) Fostamatinib, (B) Desloratadine, (C) Cromoglycate (DSCG), or (D) Formoterol and oedema formation in lung and ear tissue was assessed 30 min after intravenous DNP-BSA application. Statistical significance was calculated by 1-way ANOVA with subsequent Dunnett’s multiple comparisons test compared to vehicle-treated animals that received antibody and DNP-BSA with *p <0.05, **p <0.01 and ***p <0.001. Data are given as mean ± SEM of (n = 4–8 animals per group).

In all experiments intradermal or intratracheal application of the anti-DNP IgE antibody led to a highly significant increase in the optical density measured in the formamide extracted tissue. The SYK inhibitor Fostamatinib dose-dependently attenuated the Evans blue dye extravasation in ear and lung tissue with a maximal efficacy of 93% and 100% inhibition at the 100 mg/kg dose, respectively (p <0.001; Fig. 4A). The H_1_-receptor antagonist Desloratadine dose-dependently inhibited the ear oedema formation with a maximal effect of 92% at 10 mg/kg (p <0.001; Fig. 4B). However, in the lung Desloratadine only showed 40% inhibition of Evans blue extravasation at the highest dose (p <0.05; Fig. 4B). DSCG was ineffective in inhibiting the ear oedema formation but attenuated the lung oedema formation by 47% at the highest dose of 30 mg/kg (p <0.05; Fig. 4C). Inhaled application of the long-acting β_2_-agonist Formoterol dose-dependently reduced Evans blue dye extravasation in the lung with a maximal inhibition of 57% at the highest dose of 10 mg/ml (p <0.001; Fig. 4D). Formoterol also inhibited ear oedema formation however less potently and with a maximal effect of 37% at 10 mg/ml (p <0.01; Fig. 4D).

### 2.5. Pharmacological inhibition of antigen-induced bronchoconstriction

**Figure 5:**
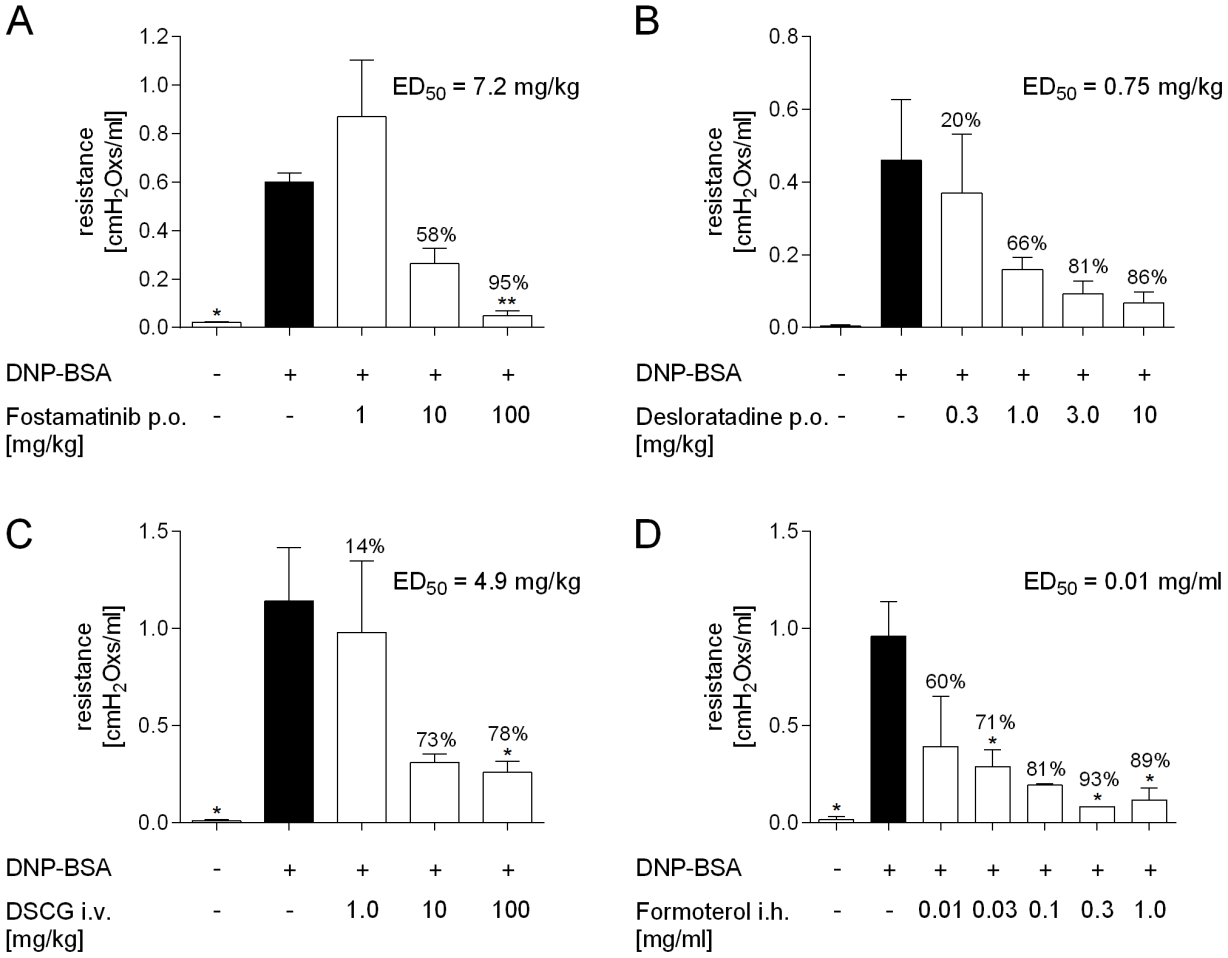
Pharmacological inhibition of antigen-induced bronchoconstriction. Rats were passively sensitized by intratracheal application with 10 µg of anti-DNP IgE antibody. The next day rats were treated with either (A) Fostamatinib, (B) Desloratadine, (C) Cromoglycate or (D) Formoterol and changes in airway resistance within 5 minutes after intravenous DNP-BSA application were recorded in anaesthetized animals. Statistical significance was calculated by 1-way ANOVA with subsequent Dunnett’s multiple comparisons test compared to vehicle-treated animals that received antibody and DNP-BSA with *p <0.05 and **p <0.01. Data are given as mean ± SEM of (n =  2–6 animals per group).

In all experiments the intravenous application of the antigen led to an increase in lung resistance which peaked within 5 minutes after DNP-BSA application as described above. The SYK inhibitor Fostamatinib dose-dependently attenuated the antigen-induced bronchoconstriction with an ED_50_ value of 7.2 mg/kg and a maximal efficacy of 95% inhibition at the highest dose of 100 mg/kg (p <0.01; Fig. 5A). The H_1_-receptor antagonist Desloratadine also dose-dependently reduced bronchoconstriction with a maximal inhibition of 86% at 10 mg/kg and an ED_50_ value of 0.75 mg/kg (Fig. 5B). Both, DSCG and Formoterol dose-dependently attenuated bronchoconstriction with ED_50_ values of 4.9 mg/kg and 0.01 mg/ml, respectively (Fig. 5C and 5D). DSCG and Formoterol showed maximal inhibition of 78% and 93% at 100 mg/kg and 0.3 mg/ml, respectively (p <0.05).

## Discussion

The aim of our study was to develop a pre-clinical *in vivo* model of passive pulmonary anaphylaxis to aid the preclinical development of novel therapeutics for asthma. Available models which are used to identify and evaluate novel therapeutic targets suffer from certain short-comings. For example many models of passive sensitization, including the well-established model of passive cutaneous anaphylaxis, are not performed in the target organ, the lung [10]. Our model demonstrated that responses observed in the ear and lung can considerably differ, thus emphasising the importance to evaluate compounds in the appropriate experimental setting. In contrast, *in vivo* models of active anaphylaxis, which are induced by the administration of antigens to animals that have been sensitized with the same agent before, are indeed commonly performed in the lung, however they are often very time-consuming, again making them inappropriate to screen for the efficacy of novel approaches [11].

In our model rats were passively sensitized by application of an IgE antibody into the lung and mast cell degranulation was induced by subsequent intravenous application of the antigen. The model reflects signs of an early asthmatic response including mast cell mediator release, lung oedema formation and bronchoconstriction as determined by the evaluation of histamine, tryptase and cysteinyl leukotriene levels in the BALF, Evans blue dye leakage into the tracheal and bronchial tissue and the increase in lung resistance. Additionally, we performed the method of passive cutaneous anaphylaxis in the same animals maximizing the information content while reducing animal numbers.

Oedema formation was more prominent in the tracheal and bronchial tissues, compared with peripheral/parenchymal lung tissue. This may reflect a higher density of mast cells in the central airways compared with the parenchymal tissue, which has been observed in healthy human tissue [12]. However, whilst overall pulmonary mast cell densities increase in patients with asthma, the proportion in the parenchyma is also increased relative to the central airways [12], and this may be one aspect of the clinical disease that is not replicated in this model. The levels of mast cell mediators in the lavage fluid were not consistent with time, with histamine levels peaking rapidly and then declining, whereas tryptase and cysteinyl leukotriene levels rose more slowly. This is consistent with the time course of the same mediators following anaphylaxis in humans, and further validates the findings described in this model [13].

To pharmacologically validate our model we tested different principals including a SYK inhibitor, a histamine H_1_-receptor antagonist, a mast cell stabilizer and a β_2_-adrenergic agonist.

The spleen tyrosine kinase, SYK, is a non-receptor tyrosine kinase which plays a key role in IgE-mediated responses in allergic asthma and rhinitis [14], [15]. Since SYK acts directly downstream of FcRI-receptors, inhibition of SYK attenuates mast cell degranulation [16]. Fostamatinib (also known as R788 or R406, its active metabolite) is an oral SYK inhibitor which was shown to attenuate mast cell degranulation in different pre-clinical settings [17]–[19]. In line with the literature Fostamatinib inhibited oedema formation in ear and lung tissue and antigen-induced bronchoconstriction in our model.

Desloratadine is a long-acting histamine H_1_-receptor inverse agonist widely used for symptomatic relief in urticaria and allergic rhinitis [20], [21]. Besides antagonizing the H_1_-recpeotor Desloratadine was also shown to attenuate the release of preformed and de novo synthesized mast cell mediators like histamine, tryptase, LTC_4_, and PGD_2_ [22]. In our model at 10 mg/kg Desloratadine inhibited ear oedema formation by 92% but at the same dose reduced lung oedema formation by only 40%. Furthermore, antigen-induced bronchoconstriction was inhibited by 86% at a dose of 10 mg/kg. The diverging effects on lung oedema formation and bronchoconstriction might possibly reflect the differing roles for histamine receptor sub-types in the lung. While the H_1_-receptor is the main receptor sub-type mediating bronchoconstriction, H_2_- and H_3_-receptors were shown to mediate vasodilatation [23], [24].

Disodium cromoglycate belongs to the group of cromones which are mast cell stabilizers that attenuate the release of mast cell mediators during the early and late asthmatic responses [25]. Despite the fact that their exact mechanism of action is largely unknown cromones are used for the routine treatment of mild to moderate asthma. In our experimental setting DSCG attenuated the lung oedema formation and bronchoconstriction with a maximal efficacy of 47 and 78%, respectively. However, the effect was only seen when DSCG was administered intravenously together with the antigen. This very short duration of action is in agreement with a study by Thomson et al. demonstrating that the maximal inhibition (98%) of DSCG on passive cutaneous anaphylaxis in rats occurred only when the drug and antigen were administered together [26]. This very short duration of action may also explain the lack of efficacy observed against ear oedema formation in the more poorly perfused skin tissue compared with the lung.

Long-acting β_2_-agonists, like Formoterol, in combination with inhaled corticosteroids (ICS) are the mainstay of current asthma medication. Short- and long-acting β_2_-agonists are primarily used because of their bronchodilatory efficacy mediated via their action on β_2_-adrenergic receptors expressed on airway smooth muscle cells. However, mast cells also express β_2_-adrenergic receptors on their surface, activation of which leads to a rapid and sustained increase in intracellular cAMP levels thereby inhibiting mast cell degranulation [27]. In accordance with this, in our model, inhalation of the β_2_-agonists Formoterol reduced antigen-induced lung oedema formation by 57% and ear oedema formation by 37% at the highest dose of 10 mg/ml. The reduced efficacy in the ear may be a consequence of the lower systemic exposure after inhalation treatment. Moreover, Formoterol attenuated the antigen-induced bronchoconstriction more potently and with a higher maximal efficacy of 93% at a dose of 0.3 mg/ml. With regard to its potency the bronchodilatory efficacy of Formoterol is probably not mediated via the inhibition of mast cell mediator release but rather directly by binding to β_2_-adrenergic receptors on the surface of airway smooth muscle cells thereby inducing smooth muscle cell relaxation and bronchodilation.

In conclusion we established a novel model of passive pulmonary anaphylaxis in rats. The model recapitulates many key features of an early asthmatic response observed in the clinics including the antigen-induced release of mast cell mediators, the subsequent formation of lung oedema and airway obstruction. The model is able to distinguish between different organ pharmacologies, is easily manageable, highly reproducible and timesaving which makes it appropriate for the testing of novel therapeutics for asthma. Finally, to validate our model we tested the efficacy of emerging and clinically relevant asthma medications which comprehensively reflected responses observed in patients with asthma.
